# Cigarette smoking among school-going adolescents in Lithuania: Results from the 2005 Global Youth Tobacco Survey

**DOI:** 10.1186/1756-0500-3-130

**Published:** 2010-05-07

**Authors:** Bradley Jamison, Adamson S Muula, Seter Siziya, Sara Graham, Emmanuel Rudatsikira

**Affiliations:** 1 Department of Global Health, School of Public Health, Loma Linda University, Loma Linda, California, USA; 2 Department of Community Medicine and Public Health, College of Medicine, University of Malawi, Blantyre, Malawi; 3 Department of Community Medicine, School of Medicine, University of Zambia, Lusaka, Zambia; 4 Division of Epidemiology and Biostatistics, Graduate School of Public Health, San Diego State University, San Diego, California, USA

## Abstract

**Background:**

The majority of people who suffer morbidity due to smoking may have initiated smoking during adolescent period. The aim of this study is to determine the prevalence and associated factors for cigarette smoking among school-going adolescents in Lithuania.

**Findings:**

Data from the Global Youth Tobacco Survey (GYTS) 2005 were used to conduct this study. Data were analyzed using SUDAAN software 9.03. Comparisons for categorical variables were done using the Pearson's Chi-square test. The cut of point for statistical significance was set at 5% level. Logistic regression analyses were conducted to determine factors associated with the outcome. Unadjusted odds ratios (OR) and adjusted odds ratios (AOR) together with their 95% confidence intervals (CI) are reported.

Of the 1822 respondents, 35.8% males and 27.1% females reported being current cigarette smokers (p < 0.001). Having friends who smoke cigarettes was associated with smoking after controlling for age, gender, parental smoking status, and perception of risks of smoking (AOR = 3.76; 95% CI [2.33, 6.90] for some friends using tobacco; and AOR = 17.18; 95% CI [10.46, 28.21] for most or all friends using tobacco). Male gender and having one or both parents who smoke cigarettes were associated with smoking (AOR = 1.31; 95% CI [1.03, 1.66]) and AOR = 1.76; 95% CI [1.37, 2.27]) respectively).

**Conclusions:**

There is a high prevalence of cigarette smoking among Lithuanian adolescents. Male adolescents and adolescents who have friends or parents who smoke should be the main target for tobacco control in Lithuania.

## Background

Tobacco use is a leading cause of adult mortality across the world. It is estimated that tobacco-attributable deaths are projected to rise from 5.4 million in 2005 to 8.3 million in 2030. By 2015, tobacco use is projected to cause 50% more deaths than AIDS [1,2].

Much of the morbidity and mortality associated with tobacco use, such as cancers, chronic lower respiratory obstructive conditions and cardiovascular morbidity and mortality are experienced after several decades of smoking [1-4]. The majority of people who suffer morbidity later in life had initiated smoking as adolescents or young adults [5].

Studies conducted in Lithuania include that of Garmiene et al [6] who reported a smoking prevalence of 1.2% among fifth grade adolescents of whom 6.5% girls and 23.0% boys had ever tried smoking. This report however was from one setting (Kaunas) and included only 369 school children. The results of the Lithuanian GYTS 2005 that have been published [7] were limited to the analysis of data for the age group 13 to 15 years; and only reported the prevalence of tobacco use. Factors associated with current smoking were not reported in this report. However, these factors have been reported elsewhere [8-10] but public health actions have to be at local, national and regional level. We thus aimed to determine the prevalence and associated factors for cigarette smoking among school-going adolescents in Lithuania.

## Methods

Our study involved secondary analysis of existing data from the Lithuania Global Youth Tobacco Survey (GYTS) conducted in 2005. A comprehensive description of the methods and procedures is presented elsewhere [7-9]. In brief, the Lithuanian GYTS conducted in 2005 was a cross sectional study, that was aimed to recruit school-going adolescents of ages 13 to 15 years using a two-stage probability sampling technique. In the first stage, primary sampling units were schools which were selected with a probability proportional to their enrolment size. In the second step, a systematic sample of classes in the selected schools was obtained. All students in the selected classes, irrespective of their actual ages (even when outside the 13 to 15 year age group) were eligible to participate. The school and class response rates were 100%. However, out of the total sample eligible for participation, 82.8% eventually participated, representing 17.2% of the eligible students who were either absent or refused to participate in the survey.

### Questionnaire and variables

A questionnaire was used and included 'core GYTS' and other additional questions as has been described elsewhere regarding the GYTS methodology [7-9]. Responses to questions were close ended with multiple-choice style format. The questionnaire included questions among others on tobacco use, knowledge and attitudes regarding tobacco, and pro- and anti-tobacco media and advertising exposure.

### Statistical analysis

For purposes of analysis, current cigarette smoking was defined as per GYTS convention which is having smoked a cigarette, even a single puff, within the last 30 days [7]. Using the socio-ecological model (SEM) of health behavior [11], we selected the variables to be analyzed. In brief the SEM recognizes that for a behavior such as adolescent smoking, various factors operating at the individual, interpersonal, organizational, structural level and policy levels interact. The questions and possible responses that were selected for analysis in this study are shown in Table 1.

**Table 1:** Questions asked and options provided in the survey, and recoding for analysis.

Question	Options provided	Re-coding for analysis
During the past 30 days (one month), on how many days did you smoke cigarettes?	0 day; 1 or 2 days; 3 to 5 days; 6 to 9 days; 10 to 19 days; 20 to 29 days; all 30 days	Any number of days except 0 were coded as current smoker = yes (1)
Do you think boys who smoke cigarettes have more or less friends?	More friends; Less friends; No difference from non-smokers	Re-coded as binary variable, less friends and makes no difference combined and recoded = 0; More friends recoded = 1
Do you think girls who smoke cigarettes have more or less friends?	More friends; Less friends; No difference from non-smokers	Re-coded as binary variable, less friends and makes no difference combined and recoded = 0; More friends recoded = 1
Do you think smoking cigarettes makes girls look more or less attractive	More attractive; Less attractive; Smoking doesn't make a difference	Less attractive and doesn't make a difference recoded = 0; More attractive coded = 1
Do you think smoking cigarettes makes boys look more or less attractive	More attractive; Less attractive; Smoking doesn't make a difference	Less attractive and doesn't make a difference recoded = 0; More attractive coded = 1
Do you think cigarette smoking is harmful to your health?	Definitely not; Probably not; Probably yes; Definitely yes	Definitely yes or probably yes coded = 1; Otherwise 0
Do any of your closest friends smoke cigarettes?	None of them; Some of them; Most of them; all of them	Indicator variables created, with one of the categories as referent
During the past 30 days (one month), when you watched sports events or other programs on TV how often did you see cigarette brand names?	I never watch TV; A lot; Sometimes; Never.	Never and I never watch TV combined and coded=0; A lot or sometimes coded = 1
During the past 30 days (one month), how many advertisements for cigarettes have you seen on billboards?	A lot; A few; None	None = 0; A lot or a few = 1
During the past 30 days (one month), how many advertisements or promotions for cigarettes have you seen in newspapers or magazines?	A lot; A few; None	None = 0; A lot or a few = 1
When you go to sports events, fairs, concerts, community events, or other events, how often do you see anti-smoking information?	A lot; A few; None	None = 0; A lot or a few = 1

Data were analyzed using SUDAAN software 9.03 (Research Triangle Institute, Research Triangle Park, North Carolina, United states of America). Comparisons for the categorical variables were statistically conducted using the Pearson's Chi-square test. The cut off point for statistical significance was set at the 5% level.

In order to estimate the associations between the explanatory variables and the outcome variable, bivariate logistic regression analyses were conducted, and obtained unadjusted odds ratios (OR) and their 95% confidence intervals (CI); and finally, a multivariate logistic regression model was run to determine independent predictors for the outcome, and the results are presented as adjusted odds ratios (AOR) and their 95% CI.

### Ethical considerations

These data were obtained on request from the Centers for Diseases Control and Prevention, Atlanta, Georgia. The study was approved by the Committee on Health Promotion of the Ministry of National Education and Religion [8]. Parents were informed of the study through a letter, and students gave verbal consent to participate in the survey. To preserve individual confidentiality, the questionnaire was self-completed anonymously by the student.

## Results

### Characteristics of study participants

A total of 1853 students participated in the survey. Of the 1822 (98.3%) students who reported their sex, 948 (52.0%) were female. The median age was 14 (Q_1 _= 13, Q_3 _= 15) years.

### Prevalence of cigarette smoking

Altogether, 35.8% males and 27.1% females reported being current cigarette smokers (p < 0.001).

Table 2 indicates that participants were exposed to tobacco advertisements through TV (70.7%), billboards (100%), and newspapers or magazines (63.4%). More than 1 in 2 respondents (54.1%) reported having seen cigarette advertisements at sports events in the past 30 days.

**Table 2:** Exposure to cigarette advertisements among adolescents in Lithuania.

Characteristics	Number of participants	% of total [95% CI*]	p-value
See cigarette adverts when watching TV	1735	70.7 [68.5, 72.9]	0.082
Males	826	73.0 [69.8, 76.0]	
Females	909	68.7 [65.5, 71.7]	
Seen cigarette adverts on billboards in past 30 days	1428	100	
Males	657	100	
Females	771	100	
Seen cigarette adverts in newspapers or magazines in past 30 days	1808	63.4 [61.2, 65.7]	0.017
Males	862	60.4 [57.1, 63.7]	
Females	946	66.2 [63.1, 69.2]	
Seen cigarette adverts at sports events in past 30 days	1800	54.1 [51.7, 56.4]	0.358
Males	861	54.9 [51.4, 58.3]	
Females	939	53.3 [50.0, 56.4]	

CI* Confidence Interval

Table 3 indicates that the vast majority (92.0%) of the respondents felt that cigarette smoking is harmful. More than two-thirds (69.5%) of the respondents thought that males who smoked cigarettes had more friends while 37.1% thought so for females. There were 1.74 times more respondents who thought that male smokers were attractive compared to those who thought so for female smokers (12.2% and 7.0%, respectively).

**Table 3:** Attitudes towards cigarette smoking distributed by gender in Lithuania.

Characteristic	Number of participants	% of total [95% CI*]	p-value
Felt that boys who smoke cigarette have more friends	832	69.5 [66.3, 72.5]	0.657
Males	391	68.4 [66.3, 72.5]	
Females	441	70.3 [66.0, 74.6]	
Felt like girls who smoke cigarettes have more friends	921	37.1 [34.0, 40.3]	0.618
Males	467	37.8 [33.5, 42.4]	
Females	454	36.3 [32.0, 40.9]	
Felt that boys who smoke cigarettes are attractive	1180	12.2 [10.4, 14.2]	0.001
Males	518	16.1 [13.1, 19.7]	
Females	662	9.1 [7.1, 11.6]	
Felt that girls who smoke cigarettes are attractive	1426	7.0 [5.8, 8.5]	0.001
Males	646	10.3 [8.2, 13.0]	
Females	780	4.3 [3.0, 6.1]	
Felt that cigarettes smoking is harmful to health	1809	92.0 [90.6, 93.2]	<0.001
Males	864	89.6 [87.3, 91.6]	
Females	945	94.1 [92.3, 95.5]	

CI* Confidence Interval

Table 4 shows that age, parental and best friend smoking status were significantly associated with current cigarette smoking in bivariate analyses. These factors remained significantly associated with the outcome in a multivariate analysis. Having friends who smoke cigarettes was very strongly associated with tobacco use after controlling for age, gender, parental smoking status, and perception of hazards caused by smoking. For those respondents who had most or all friends who smoked cigarettes, we found a more than a seventeen-fold increase in the odds of smoking compared to those who had no smoking friends (AOR = 17.18; 95% [10.46, 28.21]). Those who had some friends who smoked were more than three times likely to smoke than those who had no smoking friends (AOR = 3.76; 95% CI [2.33; 95% CI [2.33, 6.90]). Males were more likely to report cigarette smoking than females (AOR = 1.31; 95% CI [1.03, 1.66].

**Table 4:** Factors associated with current cigarette smoking in Lithuania.

Characteristic	Cigarette smokers % (n)	OR* [95% CI**]	AOR*** [95% CI]
Age (years)	31.3 (533)		
=<13	18.8 (109)	1.00	1.00
14	27.5 (167)	1.56 [1.18, 2.05]	1.16 [0.85, 1.58]
15	44.1 (203)	3.35 [2.53, 4.42]	2.22 [1.61, 3.06]
>= 16	60.3 (54)	6.04 [3.78, 9.64]	4.06 [2.37, 6.96]
Gender			
Female	27.1 (237)	1.00	1.00
Male	35.8 (284)	1.54 [1.25, 1.89]	1.31 [1.03, 1.66]
Parental smoking status			
None	22.7 (156)	1.00	1.00
One or both parents smokers	37.4 (372)	1.99 [1.60, 2.48]	1.76 [1.37, 2.27]
Best friends smoking status			
None	6.7 (21)	1.00	1.00
Some	23.9 (212)	4.35 [2.72, 6.95]	3.76 [2.33, 6.90]
Most or all	61.0 (292)	22.52 [13.94, 36.37]	17.18 [10.46, 28.21]
Perception that smoking is harmful to health			
No	35.9 (45)	1.00	1.00
Yes	30.9 (484)	0.81 [0.55, 1.18]	0.83 [0.52, 1.32]

OR* Odds ratio

CI** Confidence Interval

AOR*** Adjusted odds ratio

## Discussion

We found a prevalence of current cigarette smoking of 35.8% and 27.1% among Lithuanian school-going adolescent males and females respectively. Although there is male predominance, the smoking prevalence among females is much higher than the average prevalence of cigarette smoking among female youth in Europe [12] but similar to what has been reported in Cyprus [13]. Christophi et al [13] have reported smoking prevalence of 36% among boys and 23% among girls in high schools in Cyprus. Among in-school adolescents in some European countries, Warren et al [7] have reported prevalence of current cigarette smoking of 8.5% in Albania (2004), 26.5% in Belarus (2004), 10.4% in Greece (2005), 32.9% in Latvia (2007) and 24.1% in Croatia (2007).

The male predominance in cigarette smoking has been reported in Africa, India and Europe but is not universal [14]. A comprehensive report of global adolescent smoking patterns by Warren et al [7] has shown in general male predominance is high in Africa and Asia, while in the United States and parts of Europe, the gap between the sexes is limited. We do not know the reasons behind these patterns but we suggest that they may have to do with cultural acceptability of female smoking. If smoking among women is perceived in negative terms more than male smoking is in any particular society, we hypothesize that females in that society are less likely to smoke. The findings in the current study that about 2 in 3 adolescents reported that boys who smoke have more friends while only 1 in 3 thought that girls who smoke have more friends supports the assertion that male smokers are more accepted by society in Lithuania than female smokers.

We found that exposure to pro-tobacco advertisement exceeded half of the sample, and in some of the exposures approaching three-quarters. Data on adolescent smoking has shown that pro-teen advertisements are an important factor in influencing initiation and maintenance of adolescent smoking [4,15]. The findings that smoking in a parent, best friend and increasing age were positively associated with smoking have also been reported elsewhere [16-19].

This study has several inherent limitations. Firstly, the data were collected via a self-reported questionnaire. Like all questionnaires, the possibility of mis-reporting both intentional and unintentional threatens the validity and reliability of the findings. No biomarkers were assessed to confirm current cigarette smoking status. However, data from the United States using a similar questionnaire as the GYTS have reported high reliability among students in reporting personal health-compromising behaviors [20,21]. The extent as to whether similarly high reliability values could be obtained in settings outside of the United States is not known. Secondly, only students enrolled and available in schools during the administration of the questionnaire and completed it, were surveyed; leaving out 17.2% of adolescent students. To the extent that these students are not representative of all the adolescents in school, and of the overall adolescent population in the country (including out of school adolescents) our findings may not be generalized to the in-school adolescents, and to the adolescent population in Lithuania.

## Conclusions

Our study has found that the prevalence of cigarette smoking among adolescent students was 35.8% for males and 27.1% for females. Being male and having friends or parents who smoke were associated with cigarette smoking. Male adolescents and adolescents who have friends or parents who smoke should be the main target for tobacco control guided by the WHO Framework Convention on Tobacco Control that Lithuania ratified in 2004.

## Competing interests

The authors declare that they have no competing interests.

## Authors' contributions

BJ participated in the interpretation of data and drafting of the manuscript; ASM participated in the interpretation of the results and led the manuscript drafting effort. SS participated in the interpretation of the results and the drafting of the manuscript. SG participated in the interpretation of data and drafting of the manuscript; and ER designed the data analysis plan, conducted the analysis and participated in the interpretation of the results. All authors read and approved the final manuscript.
